# Supplementary material for: Neuronal morphologies built for reliable physiology in a rhythmic motor circuit
Source: eLife. 2019 Jan 18;8:e41728. doi: 10.7554/eLife.41728 (PMC6349406; doi:10.7554/eLife.41728)
Supplement: Transparent reporting form [file elife-41728-transrepform.pdf]

eLIFE
1st Floor
24 Hills Road
Cambridge CB2 1JP, UK
P 01223 855340
W elifesciences.org
T @elife

eLife's transparent reporting form

We encourage authors to provide detailed information *within their submission* to facilitate the interpretation and replication of experiments. Authors can upload supporting documentation to indicate the use of appropriate reporting guidelines for health-related research (see EQUATOR Network), life science research (see the BioSharing Information Resource), or the ARRIVE guidelines for reporting work involving animal research. Where applicable, authors should refer to any relevant reporting standards documents in this form.

If you have any questions, please consult our Journal Policies and/or contact us: editorial@elifesciences.org.

Sample-size estimation

- You should state whether an appropriate sample size was computed when the study was being designed
- You should state the statistical method of sample size computation and any required assumptions
- If no explicit power analysis was used, you should describe how you decided what sample (replicate) size (number) to use

Please outline where this information can be found within the submission (e.g., sections or figure legends), or explain why this information doesn't apply to your submission:

Experiments. In this study, glutamate photo-uncaging experiments were executed in 4 different neuron types (N = 5-6 neurons of each type). Within each neuron, glutamate responses were evoked at 10-30 distinct sites, on 3-4 distinct branches of the neurite tree. Due to the technical difficulty of these experiments and based on similar experiments conducted in Otopalik et al., 2017b, these sample sizes sufficiently represent the variability within each cell type, across animals. However, because the sample size for individual neurons is < 10, the raw data for individual neuron types can be found in the Figure supplements (to Figures 3 and 5), where appropriate.

Computational Simulations. For simulations of electrotonus, direction-sensitivity, and voltage summation arithmetic, a library of 720 cable models spanning a broad parameter space were assessed. There were no statistical tests conducted on these data as these are intended to be proof-of-concept simulations, but parameter ranges and methods are described in the Materials and Methods and associated figure legends.

Replicates

- You should report how often each experiment was performed
- You should include a definition of biological versus technical replication
- The data obtained should be provided and sufficient information should be provided to indicate the number of independent biological and/or technical replicates
- If you encountered any outliers, you should describe how these were handled
- Criteria for exclusion/inclusion of data should be clearly stated

- High-throughput sequence data should be uploaded before submission, with a private link for reviewers provided (these are available from both GEO and ArrayExpress)

Please outline where this information can be found within the submission (e.g., sections or figure legends), or explain why this information doesn't apply to your submission:

All sample sizes, as discussed above, are reported in the text and/or figure legends. Definitions of photo-stimulation trials are discussed in the Methods in the "Electrophysiology Analysis" section. No outliers were excluded from the data set.

eLife Sciences Publications, Ltd is a limited liability non-profit non-stock corporation incorporated in the State of Delaware, USA, with company number 5030732, and is registered in the UK with company number FC030576 and branch number BR015634 at the address 1st Floor, 24 Hills Road, Cambridge CB2 1JP | August 2014

Statistical reporting

- Statistical analysis methods should be described and justified
- Raw data should be presented in figures whenever informative to do so (typically when N per group is less than 10)
- For each experiment, you should identify the statistical tests used, exact values of N, definitions of center, methods of multiple test correction, and dispersion and precision measures (e.g., mean, median, SD, SEM, confidence intervals; and, for the major substantive results, a measure of effect size (e.g., Pearson's r, Cohen's d)
- Report exact p-values wherever possible alongside the summary statistics and 95% confidence intervals. These should be reported for all key questions and not only when the p-value is less than 0.05.

Please outline where this information can be found within the submission (e.g., sections or figure legends), or explain why this information doesn't apply to your submission:

Analytical and statistical methods were primarily used to assess the experimental data, as the computational simulations are to be interpreted as a proof of concept. Referring to the experimental data presented:

Figure 3 and Supplements. As shown in Figure 3B, linear regressions were used to calculate apparent reversal potentials (E_{rev}s) for individual photo-stimulation sites, as in Otopalik et al., 2017b. For all apparent E_{rev}s used in these analyses, p-values < 0.05 and R values > 0.85; this is described in the Methods (Electrophysiology Analysis), but not the main focus of this manuscript as the approach is described in great detail in Otopalik et al., 2017b. Linear regression analyses were also used to: 1) demonstrate the insignificant linear relationships between response amplitudes and distance of photo-stimulation site or 2) demonstrate the near-zero slopes of the linear regressions of apparent reversal potential as a function of distance of photo-stimulation site. The results of these statistical analyses are listed in Table 1. Because these data were collected in fewer than 10 neurons of each type (yet, numerous sites within individual neurons), the raw response amplitudes and apparent reversal potentials for individual sites, for each neuron, are shown in Supplements 1-4 to Figure 3 (Parts A, C). Coefficients of variance (CVs) for apparent E_{rev}s were calculated across sites within individual neurons and mean CVs were calculated across neurons of the same type to address variability across animals. These are listed in Table 2. These approaches are described in the main text and figure legends. It should be noted that these analytical approaches are consistent with Otopalik et al., 2017b, wherein there is an in-depth discussion of their rationale.

Figures 5, 6 and Supplements. Directional biases (or sensitivity) were assessed by comparing responses in the inward and outward directions on single branches. To do so, responses in either direction were scatter plotted against each other and a measure of sensitivity was determined by fitting these data to the identity line and calculating the root mean squared error (RMSE). The same method was used to assess arithmetic of voltage integration in single branches. This approach is described in the Materials and Methods, the associated figure legends, and the main text.

(For large datasets, or papers with a very large number of statistical tests, you may upload a single table file with tests, Ns, etc., with reference to sections in the manuscript.)

Group allocation

- Indicate how samples were allocated into experimental groups (in the case of clinical studies, please specify allocation to treatment method); if randomization was used, please also state if restricted randomization was applied
- Indicate if masking was used during group allocation, data collection and/or data analysis

Please outline where this information can be found within the submission (e.g., sections or figure legends), or explain why this information doesn't apply to your submission:

For experimental work, measures of the apparent reversal potentials, response amplitudes, directional biases, and summation arithmetic were grouped by neuron type.

To compare reversal potentials and maximal response amplitudes across multiple neurons of the same type, these metrics for individual sites were normalized to the mean within the individual neuron, these normalized values for all neurons of the same type were then plotted on the same axes (as shown in Figures 3C, D and parts B and D of Supplements to Figure 3). These is described in the main text and associated figure legends.

To compare directional biases and summation arithmetic across multiple neurons of the same type, these metrics for individual branches, across multiple neurons, were plotted on the same axes and fit to the identity line (therefore requiring no normalization of these metrics for branches within the same neuron). These grouped data can be found in Figures 5, 6, and Supplements to Figure 5 (Parts E-G).

Additional data files ("source data")

- We encourage you to upload relevant additional data files, such as numerical data that are represented as a graph in a figure, or as a summary table
- Where provided, these should be in the most useful format, and they can be uploaded as "Source data" files linked to a main figure or table
- Include model definition files including the full list of parameters used
- Include code used for data analysis (e.g., R, MatLab)
- Avoid stating that data files are "available upon request"

Please indicate the figures or tables for which source data files have been provided:

All source experimental and simulated data, as well as associated computational scripts for analysis and simulations, will be made available on the Marder Lab GitHub upon publication of this study. We are currently in the process of preparing these data and scripts for upload onto this publically-available site.

eLife Sciences Publications, Ltd is a limited liability non-profit non-stock corporation incorporated in the State of Delaware, USA, with company number 5030732, and is registered in the UK with company number FC030576 and branch number BR015634 at the address 1st Floor, 24 Hills Road, Cambridge CB2 1JP | August 2014
